# Taking the path of least resistance now, but not later: Pushing cognitive effort into the future reduces effort discounting

**DOI:** 10.3758/s13423-022-02198-7

**Published:** 2022-11-04

**Authors:** S. Tobias Johnson, Steven B. Most

**Affiliations:** School of Psychology, University of New South Wales, Sydney, NSW 2052 Australia

**Keywords:** Cognitive effort, Effort discounting, Delay discounting, Procrastination, Dynamic inconsistency, Need for cognition, Individual differences

## Abstract

*Effort discounting* describes the devaluation of rewards that require effort to obtain. The present study investigated whether discounting of cognitive effort depends on how near the effort is in time. The present study also investigated whether effort discounting, and its modulation by temporal distance to the effort, might depend on *need for cognition*, a personality trait that describes how much one enjoys cognitively demanding tasks. Participants performed a validated effort discounting task that measured the extent to which they subjectively devalued a $20 reward when effort was required to receive it. Immediacy of the effort was manipulated by having participants imagine exerting varying levels of effort either immediately, in a day, or in a month. Results revealed linear increases in discounting of rewards as a function of both how much effort was involved and how imminent the effort was. The extent to which both these variables influenced discounting correlated with need for cognition. Individuals low in need for cognition exhibited more effort discounting overall and a linear increase in effort discounting as the effort grew imminent. Individuals high in need for cognition engaged in less effort discounting, which was not modulated by how imminent the effort was. These results indicate that people exhibit dynamic inconsistency in effort-related decisions, such that the degree to which they discount effort depends on how soon the effort is. Additionally, this tendency is linked with systematic individual differences in need for cognition. Lastly, this study demonstrates that these tendencies can be quantitatively operationalized.

Effort is a cost factor that can modulate the degree to which we subjectively value rewards. When viewed in the rear-view mirror (i.e., when it is already expended), effort can increase how valuable a reward is perceived to be (Norton et al., 2012). But when people *anticipate* exerting effort, the appeal of the reward often depreciates. This depreciation of subjective value is known as *effort discounting* (Botvinick et al., 2009), and it is a phenomenon familiar to many through first-hand experience. For example, although many of us may aspire to improve our physical fitness, the effort required to achieve this goal can make the process difficult to commit to.

Experiments seeking to understand subjective valuation frequently use tasks that operationalize discounting as the degree to which preference for an option decreases in the presence of a cost factor (e.g., delay, effort, or uncertainty; Białaszek et al., 2019). Of these, “delay discounting” is perhaps the most widely known and studied. In such a task, participants choose between options that have different objective values, and researchers assess how choices change along with varying durations of anticipated delay (Mazur, 1987). For example, when asked to choose between receiving $10 or $20, both of which are immediately available, most people will choose the objectively larger amount; but when choosing between $10 now and $20 next year, many people choose the smaller, more immediate amount. Such tasks provide a way to investigate modulators of subjective value more objectively than can be achieved through self-report alone (Westbrook et al., 2013).

Effort discounting has been investigated in a similar way. In one experiment, for example, participants proceeded through a series of choices between a smaller reward that required low effort and a larger reward that required more effort (Libedinsky et al., 2013). Throughout the series of decisions, the larger reward was fixed (e.g., at $20) while the smaller reward was adjusted up or down. The aim was to reveal the participant’s *indifference point*, which is the objective value of the smaller reward at which the participant shows no clear preference between the smaller, effortless reward and the larger, effortful reward (also see Mazur, 1987). As a concrete example, if a person displays no clear preference between expending no effort to receive $12 and expending significant effort to receive $20, their indifference point in the face of the extra effort is $12, the point at which the *subjective* value of each option is equal. In this way, the indifference point provides an objective measurement, in dollars, of the subjective value of the numerically larger reward once anticipated effort is taken into account.

The delay discounting literature contains clues as to how effort discounting might change as people anticipate the effort to lie further in the future. Typically, models of optimal decision making assume that rational individuals are dynamically consistent, meaning they have consistent preferences throughout time. That is, such models may assume that if a person prefers A to B at a certain point in time, they will prefer A to B at all other points in time (Read et al., 1999). However, delay discounting experiments have shown that people display *dynamic inconsistency*, whereby preferences change if the options are pushed out in time in equal amounts (Green et al., 1981; Green et al., 1994). For example, if choosing between $50 now versus $100 in 6 months (a 6-month difference), it may be tempting to choose $50 now. But if the two choices are pushed forward in time, so that the choice is between $50 in 3 months or $100 in 9 months (also a 6-month difference), the delay is now discounted to a lesser degree; the $50 is not as tempting and one’s preference may switch to the $100 option (see Read & van Leeuwen, 1998, for an example related to eating behaviour). It seems that when delay is the cost factor, discounting occurs at a higher rate when the anticipated rewards are more imminent.

It may be that people are also dynamically inconsistent when effort is the cost factor. For example, people may *intend* to perform a difficult task in service of a desired outcome (“Next Monday, I’m going to finish my essay”), but as the effort draws nearer in time, their motivation may wane, leading them to opt for an easier but less desired outcome. Dynamic inconsistency in the context of effort discounting may contribute to the gap between intention and action so typical of human behaviour. Indeed, in one study, participants were assigned a number of effortful tasks, which they were allowed to distribute across two weeks; when distributing the tasks between 2 and 3 weeks in the future, they allocated more tasks to the earlier week compared to when they were asked to distribute the tasks between 1 and 2 weeks in the future (Augenblick et al., 2015). This suggests that dynamic inconsistency occurs in the context of effort discounting.

## The current study

The present study aimed to build on previous work showing that people discount options that require effort (e.g., Libedinsky et al., 2013; Westbrook et al., 2013), with participants typing a varying number of words backwards as the manipulation of cognitive effort (e.g., Libedinsky et al., 2013). In addition to replicating this earlier finding, a second, novel aim was to determine whether indifference points (as a measure of the magnitude of effort discounting) change when anticipated effort is pushed into the future. Finally, we tested whether individual differences in the degree to which people discount cognitive effort would emerge as a function of “need for cognition,” a self-report measure designed to index the enjoyment people get from cognitive challenge (Cacioppo & Petty, 1982). Specifically, it was predicted that high need for cognition would be associated with less discounting of cognitive effort.

## Method

### Participants

One-hundred-and-one non-prescreened first-year undergraduate psychology students at the University of New South Wales participated in this web-based study in return for course credit. Sample size was larger than needed for the medium to large effect sizes previously associated with main effects of effort discounting (e.g., Westbrook et al., 2013); to accommodate the likely subtler modulating effects of temporal distance and need for cognition targeted by this study, we recruited more participants than suggested by G*Power (Faul et al., 2007). Due to an oversight, the age and sex of participants were not recorded. The invitation to participate was extended to students registered in the UNSW experiment participation system; participants provided informed consent, and the study was approved by the Human Research Ethics Advisory Panel in the School of Psychology. Data from 29 participants were discarded because at least one trial had either a reaction time of less than 100 ms or a Levenshtein distance of backwards typing task input greater than 40 (Levenshtein, 1966). The Levenshtein distance is the minimum number of edits (insertions, deletions, or substitutions of a single character) that must be made to turn one string into another, and this was used as a measure of typing accuracy.

### Materials and design

The experiment was programmed in JavaScript using the jsPsych library (de Leeuw, 2015), and hosted online with JATOS (Lange et al., 2015). The experiment took approximately 20 minutes to complete. Need for cognition of each participant was measured using an 18-item Need for Cognition Scale, in which scores can range from −72 to +72 (Cacioppo & Petty, 1982; Cacioppo et al., 1984). Items on the Need for Cognition Scale include questions such as “I would prefer complex to simple problems,” “Thinking is not my idea of fun,” and “I find satisfaction in deliberating hard and for long hours.” For each item, participants indicate the degree to which they agree with the statement on a scale ranging from +4 (very strong agreement) to −4 (very strong disagreement).

Anticipated effort level (i.e., the number of words people imagined typing backwards) and temporal distance (i.e., how far in the future the effort would be exerted) were the factors of a 4 (effort level: 25 vs. 50 vs. 75 vs. 100 words) × 3 (temporal distance: now vs. in 1 day vs. in 1 month) within-subjects design. For all participants, the temporal distance increased in order of delay from “now,” to “in a day,” to “in a month,” cycling through all the effort levels at each temporal distance before moving to the next temporal distance. The order of effort levels for each participant was randomized, but this order was held constant for each temporal distance condition.

### Procedure

Participants first completed the Need for Cognition Scale (Cacioppo & Petty, 1982; Cacioppo et al., 1984). Then, to familiarize participants with the effortful task that they would be queried about later in the experiment, participants were shown a series of 50 words, which they were asked to type backwards. After familiarizing themselves with the backwards typing task, participants completed a series of questions that each involved choosing between a smaller, easier reward and a larger, harder reward, based on a task developed by Libedinsky et al. (2013). Before the questions, participants were informed that their choices were potentially real, and that one of their choices may be chosen at random. They were informed that if this occurred, they would be provided with a shopping voucher at the value of their answer, provided they were willing to perform the backwards typing task.

**Fig. 1:**
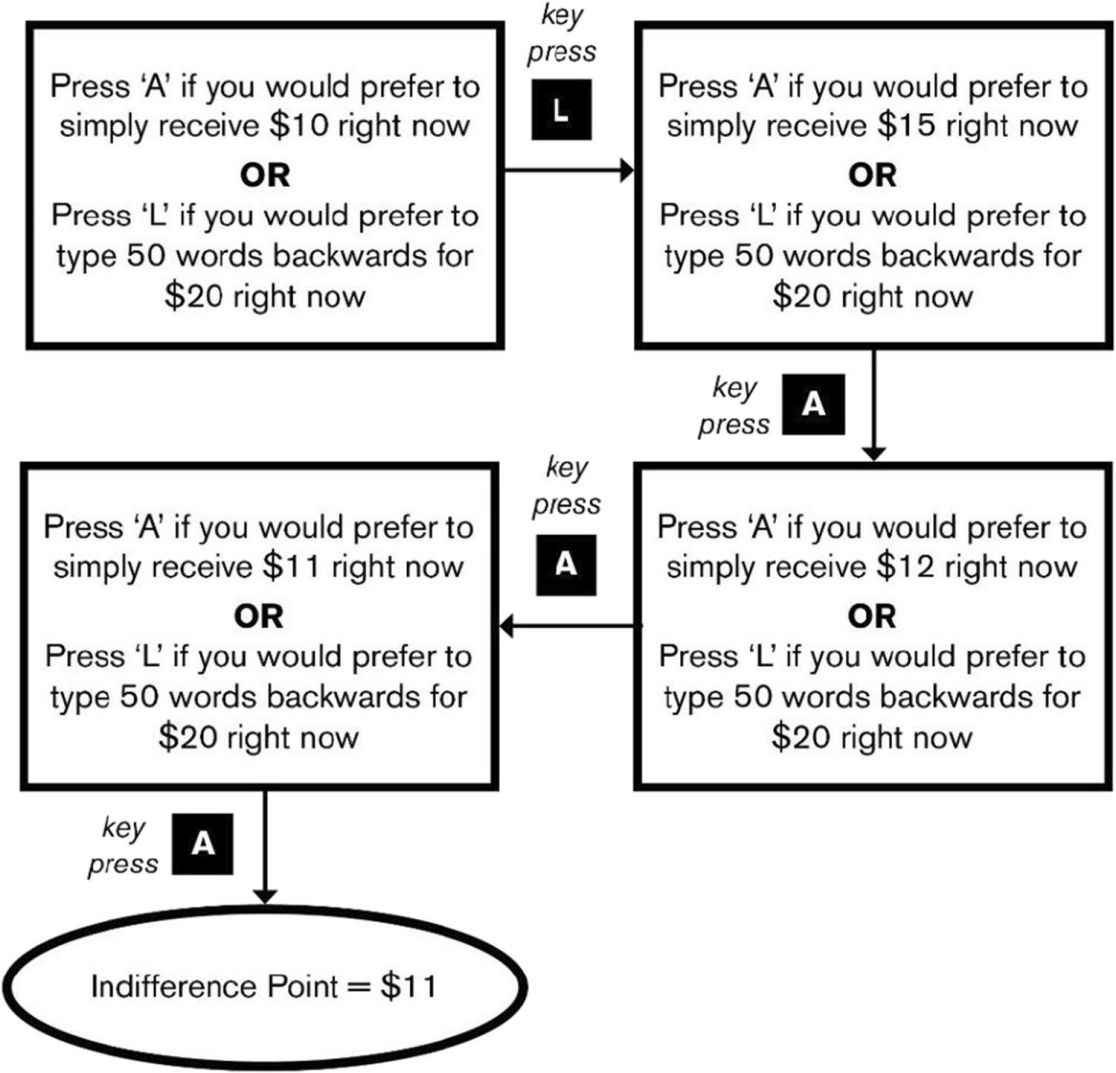
Hypothetical example of finding the subjective value of typing 50 words immediately for $20. Indifference point is the index of subjective value. The value of the smaller, easier reward was adjusted via a binary search algorithm based on participant response. In this example, the binary search algorithm stopped because the participant preferred the larger, harder reward when the smaller, easier reward was $10, but when the smaller, easier reward was just $1 different ($11), the participants preference changed to the smaller, easier reward. Therefore, the participant is said to be indifferent between the rewards when the smaller, easier reward is $11

The level of effort associated with the reward was manipulated by adjusting the number of words that would need to be typed backwards to receive it: While the smaller, easier reward required no words to be typed, the larger, harder reward required either 25, 50, 75, or 100 words to be typed, depending on the condition. For example, one question read, “Would you prefer to receive $12 in a month, or type 75 words backwards for $20 in a month?” The larger, harder reward had a fixed value of $20 throughout the experiment. At the start of each effort level condition, the value of the smaller, easier reward was randomized to be between $7 and $12. Only whole-dollar values were used throughout the experiment. To find the indifference point (and thus subjective value), participants’ answers to each question caused the value of the smaller, easier reward to change: If the participant preferred the smaller, easier reward, the value of the smaller, easier reward was lower in the next question. If the participant preferred the larger, harder reward, the value of the smaller, easier reward was higher in the next question. The direction and magnitude of the change in the smaller, easier reward was determined by a binary search algorithm: At the start of each effort level condition, an array of ascending numbers from 1 to 20 was generated. If the participant preferred the larger, harder reward, the binary search algorithm removed all numbers in the array below the value of the current smaller, easier reward. The median of the new array became the smaller, easier reward in the next question. On the other hand, if the participant preferred the smaller, easier reward, all numbers above the value of the current smaller, easier reward were removed from the array, and the median of the new array became the new smaller, easier reward in the next question. Whenever the number of items in the array was even, the smaller of the middle two numbers in the array was selected upon the participant’s response instead of the median. This ensured that the smaller, easier reward in the next trial was always a whole number. As a result of this algorithm, the amount by which the smaller, easier reward changed grew smaller on each trial. The binary search algorithm stopped when the participant’s preference between the two rewards on a given trial could be reversed by as little as a $1 change. A schematic of this process is shown in Fig. 1.

Temporal distance was manipulated by altering the wording of questions in the effort selection task so that the reward was received and the associated effort was exerted either “now,” “in a day,” or “in a month.” At each temporal distance category, indifference points were derived at all four effort levels before moving to the next temporal distance. The indifference points for each temporal distance and effort level were used as a measure of subjective value.

### Statistical analyses

Statistical analyses were conducted using IBM SPSS Statistics (Version 26). For repeated-measures analyses of variance (ANOVA) tests, the degrees of freedom were adjusted where the assumption of sphericity had been violated (according to Mauchly’s test of sphericity), by applying either the Greenhouse–Geisser correction (if ε ≤ .75) or the Huynh–Feldt correction (if ε > .75; Huynh & Feldt, 1976).

#### Effort discounting and temporal distance

A 3 (temporal distance) × 4 (effort level) repeated-measures ANOVA was planned in order to assess (a) whether effort discounting is mitigated as options are pushed out into the future (as would be reflected in a main effect of temporal distance), (b) whether effort discounting occurs along a gradient such that higher levels of effort are associated with more discounting (as would be reflected in a main effect of effort level), and (c) whether pushing effort into the future modulates sensitivity to gradation of effort (as would be reflected in a two-way interaction). Follow-up contrasts and two-tailed *t* tests were planned in order to provide further insight into what might drive effects that emerged from the omnibus ANOVA.

#### Effort discounting and need for cognition

To replicate a finding from Westbrook et al. (2013), that trait *need for cognition* correlates positively with subjective value (and thus negatively with effort discounting), a Pearson correlation was planned between subjective value (averaged across temporal distance and effort level) and score on the Need for Cognition Scale.

To investigate whether need for cognition modulated the degree to which effort discounting changed as one imagined delaying the effort, a Pearson correlation was planned between need for cognition and the difference in subjective value (averaged across effort levels) between the most distant temporal distance (one month) and the most immediate temporal distance (now). A similar analysis was planned to investigate whether need for cognition predicted sensitivity of subjective value to gradations in effort level. In this instance, a Pearson correlation was planned between need for cognition and the difference in mean subjective value between the highest effort level (typing 100 words backwards) and lowest effort level (typing 25 words backwards).

In the event these correlation analyses yielded statistically meaningful patterns, follow-up ANOVAs, contrasts, and *t* tests were anticipated, as appropriate, in order to better understand the nature of these individual differences.

## Results

### Effort discounting, temporal distance, and sensitivity to gradations of effort

**Fig. 2:**
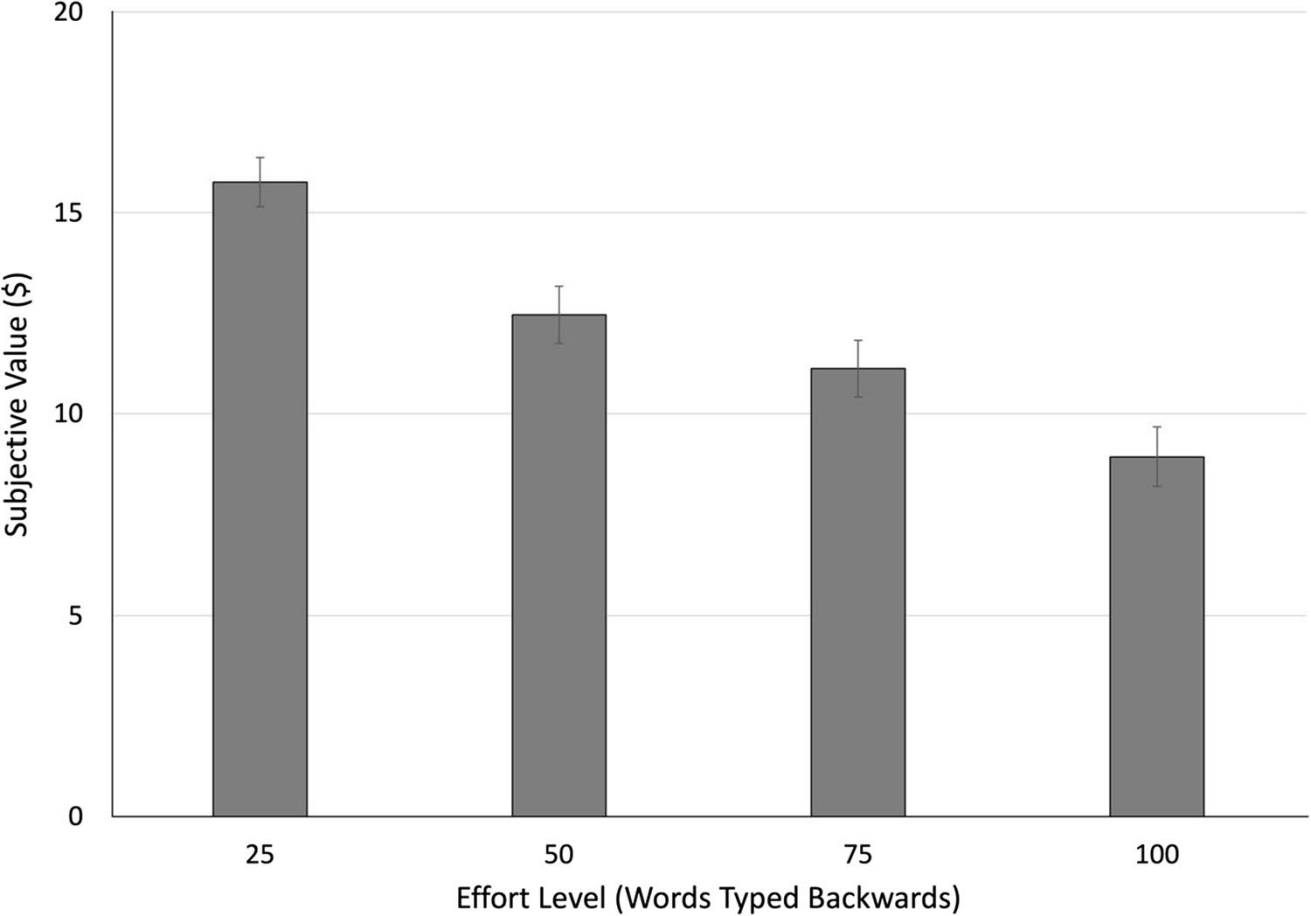
Subjective value of a reward is discounted with increasing levels of effort. The reward had an objective value of $20, but subjective value—operationalized as the indifference point—was lower as levels of effort increased. Error bars depict standard errors of the mean

The 3 (temporal distance) × 4 (effort level) within-subjects ANOVA revealed a significant main effect of effort level, consistent with the prediction that increasing levels of effort were associated with lower subjective value (i.e., more effort discounting), *F*(2.0, 142.0) = 76.21, *p* < .001, η_p_^2^ = .518 (see Table 1). A significant linear trend indicated that the change in effort discounting across effort levels followed a relatively stable trajectory, *F*(1, 71) = 123.22, *p* < .001, η_p_^2^ = .634 (see Fig. 2), though the presence of a cubic trend suggested that this trajectory was not perfectly linear, *F*(1, 71) = 13.23, *p* = .001, η_p_^2^ = .157. A test for a quadratic trend did not reach statistical significance, *F*(1, 71) = 3.24, *p* = .08, η_p_^2^ = .044.

**Table 1:** Subjective value of a $20 reward when its receipt requires exertion of varying degrees of effort either now, in 1 day, or in 1 month’s time

		Subjective value ($)	
Temporal distance	Effort level	Mean	Std. dev.
Now	25 words	15.39	5.46
	50 words	11.19	6.90
	75 words	9.97	6.62
	100 words	7.63	6.56
1 Day	25 words	15.86	5.56
	50 words	13.15	6.30
	75 words	11.44	6.50
	100 words	9.04	6.60
1 Month	25 words	16.04	5.33
	50 words	13.03	6.34
	75 words	11.94	6.42
	100 words	10.17	6.66

Temporal distance refers to how far away in time the reward and its associated effort were to be received or exerted, respectively. Effort level refers to the number of words that had to be typed backwards to receive the larger, harder reward.

**Fig. 3:**
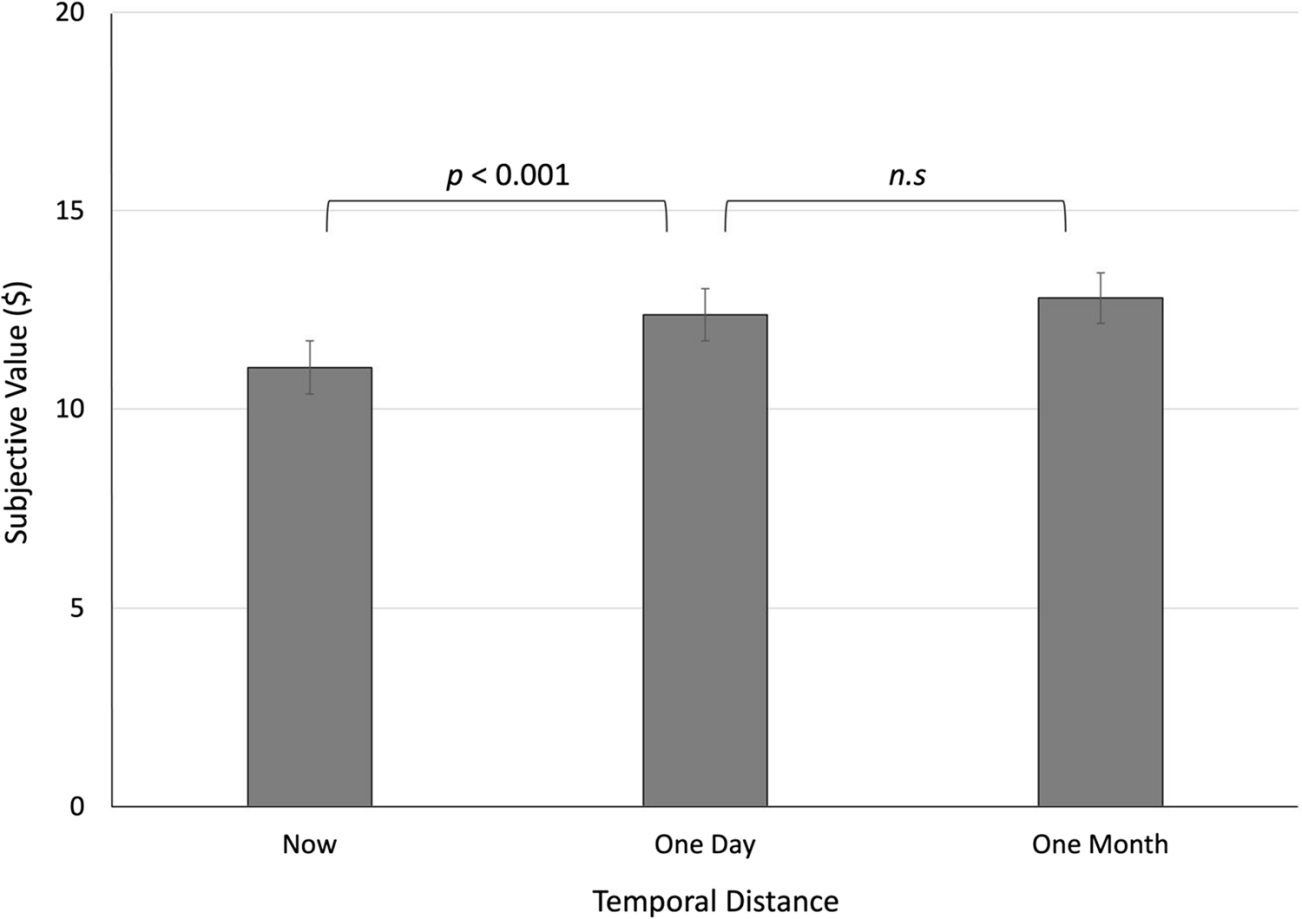
Subjective value of an effortful reward increases if the associated effort lies in the future. The reward had an objective value of $20, but subjective value—operationalized as the indifference point—was lower the sooner that participants anticipated exerting effort. Error bars represent standard errors of the means

A main effect of temporal distance emerged, consistent with the prediction that effort discounting would decrease (i.e., the subjective value would increase) as effort was imagined to lie further in the future, *F*(1.5, 108.5) = 15.14, *p* < .001, η_p_^2^ = .176. A significant linear trend indicated that the change in effort discounting across points in time followed a relatively stable trajectory, *F*(1, 71) = 17.87, *p* < .001, η_p_^2^ = .201. However, the presence of a quadratic trend suggested a curvilinear quality to this trajectory, *F*(1, 71) = 5.63, *p* = .02, η_p_^2^ = .073; and indeed, as observed in Fig. 3, there appeared to be a greater difference in effort discounting between the “now” and “in a day” time points, *t*(71) = 4.33, *p* < .001, *d* = .51, 95% CI [.71, 1.94], than between the “in a day” and “in a month” time points *t*(71) = 1.65, *p* = .104, *d* = .19, 95% CI [−.09, .90].

An effort level by temporal distance interaction also emerged, consistent with the prediction that sensitivity to gradations in effort level would diminish as effort was imagined to lie further in the future, *F*(5.5, 387.8) = 2.82, *p* = .013, η_p_^2^ = .038.

### Effort discounting and need for cognition

**Fig. 4:**
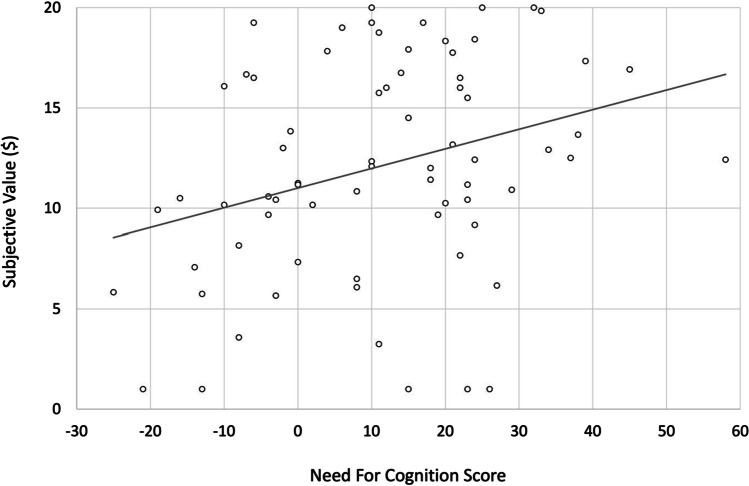
Need for cognition is related to effort discounting. Mean subjective values (of an objective $20), averaged across temporal distance conditions and effort level, displayed as a function of individual differences in need for cognition

Replicating previous work (Westbrook et al., 2013), a significant correlation indicated that need for cognition score was positively related to subjective value in the face of effort, *r*(70) = .310, *p* = .008. In other words, the higher one’s self-reported need for cognition, the less effort discounting occurred (see Fig. 4).^1^

### Need for cognition and sensitivity to gradations in either temporal distance or effort

Additional Pearson correlations assessed whether need for cognition correlated with the extent to which subjective value changed (a) as options were made more immediate and (b) as effort levels increased. Towards this end, difference scores were created between the most extreme levels of each factor (i.e., subjective value at the most immediate point in time [now] vs. at the most distant point in time [1 month], and subjective value when effort involved typing 100 words backwards vs. 25 words backwards). The correlation between sensitivity to temporal distance and need for cognition was statistically significant, *r*(70) = .276, *p* = .019. The correlation between sensitivity to gradations of effort level and need for cognition was non-significant, *r*(70) = .072, *p* = .547.

To further understand how need for cognition modulated the effect of pushing anticipated effort into the future (as indicated by the significant correlation with sensitivity to temporal distance), an additional analysis compared participants falling within the highest third of need for cognition scores (*N* = 24, *M* = 28.96, *SD* = 9.12) with those falling within the lowest third of need for cognition scores (*N* = 24, *M* = −8.13, *SD* = 7.04). Those falling within the middle third of need for cognition scores were excluded from this analysis in order to ensure a meaningful difference between groups (e.g., minimizing the possibility that group assignment was muddied by measurement noise from self-report). Data were submitted to a mixed 2 (group: high vs. low need for cognition) × 3 (temporal distance: now vs. 1 day vs. 1 month) ANOVA. Critically, the interaction between temporal distance and need for cognition was statistically significant, *F*(1.70, 78.15) = 3.92, *p* = .030, η_p_^2^ = .078.

**Fig. 5:**
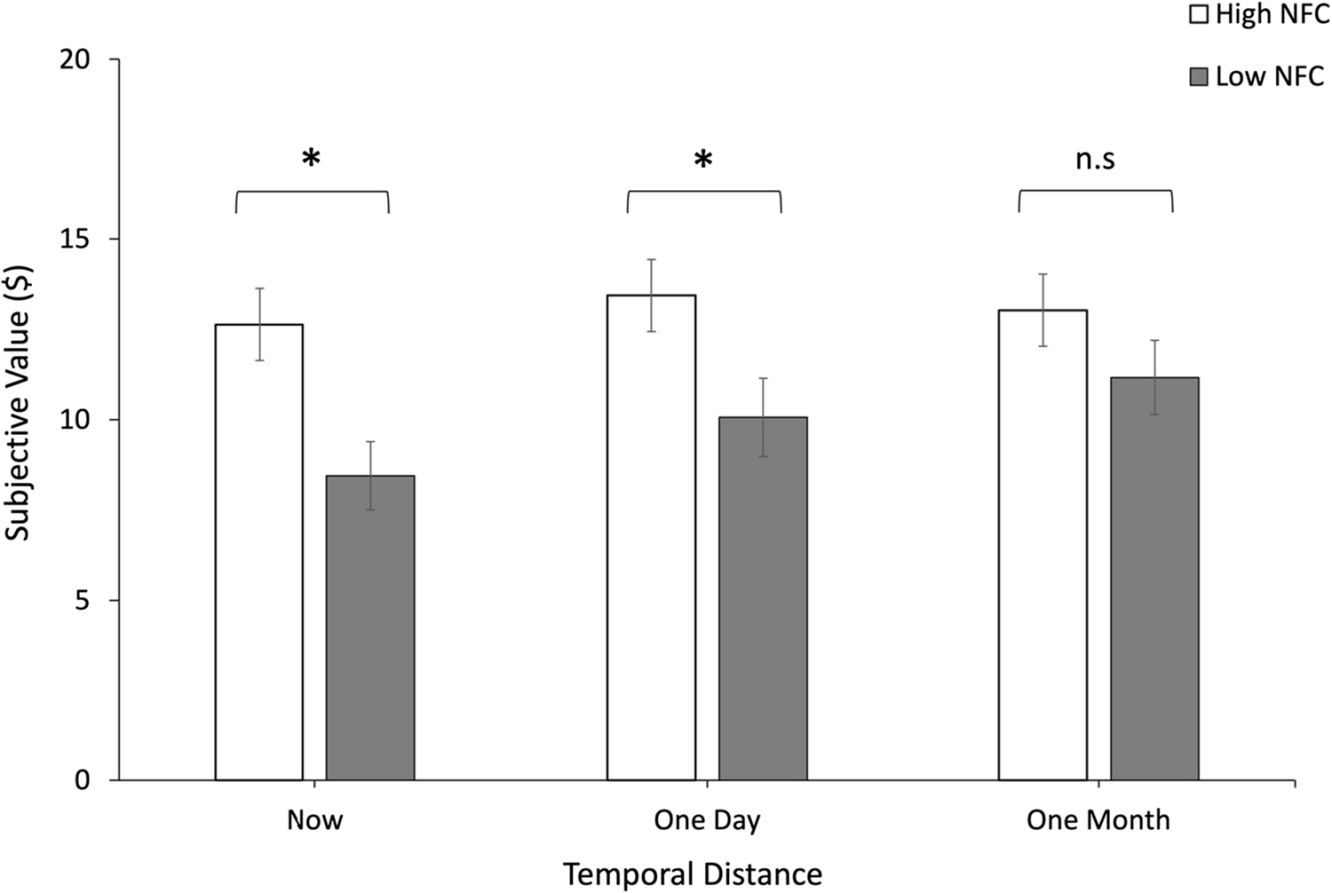
Temporal distance increases the similarity in effort discounting between people high and low in need for cognition (NFC). Mean subjective values (of an objective $20) are displayed. Error bars represent standard errors. *p < .05

The pattern in Fig. 5 suggests that the interaction between need for cognition and temporal distance emerged because people high in need for cognition were less likely to engage in effort discounting regardless of temporal distance, whereas those low in need for cognition exhibited effort discounting in the face of immediate effort, which dissipated as effort was pushed into the future. Indeed, follow-up *t* tests revealed that the high and low need for cognition groups differed in their degree of effort discounting in the face of immediate effort, *t*(46) = 2.78, *p* = .008, *d* = .80, 95% CI [1.16, 7.22]; high need for cognition: *M* = 12.64, *SD* = 5.74; low need for cognition: *M* = 8.45, *SD* = 4.63, and when effort was anticipated after a day’s delay, *t*(46) = 2.17, *p* = .036, *d* = .63, 95% CI [.237, 6.51]; high need for cognition: *M* = 13.44, *SD* = 5.51; low need for cognition: *M* = 10.06, *SD* = 5.29. In contrast, when effort was pushed one month into the future, the two groups did not differ as substantially (*t* = 1.21, *p* = .234, *d* = .35, 95% CI [−1.25, 4.98]; high need for cognition: *M* = 13.03, *SD* = 5.67; low need for cognition: *M* = 11.17, *SD* = 5.03).^2^ (An additional Bayesian *t* test at the 1-month time point, using the default prior in JASP, revealed anecdotal evidence for the null: BF_10_ = .52). Consistent with this interpretation, within-subjects contrasts revealed that the pattern of subjective value with increasing temporal distance among those scoring high in need for cognition followed neither a linear nor a quadratic trend (*p* = .64, η_p_^2^ = .010 and *p* = .10, η_p_^2^ = .114, respectively), whereas a strong linear trend emerged among the low scorers, indicating that they discounted effort progressively less as the effort was pushed into the future, *F*(1, 23) = 20.97, *p* < .001, η_p_^2^ = .477. There was no significant quadratic trend, *p* = .518, η_p_^2^ = .018.

### Additional exploratory analyses: Relationship between performance and discounting

To assess whether participants’ effort discounting reflected the degree to which they found the backwards typing task difficult, we ran a Pearson correlation between task error (the “Levenshtein distance”) and average subjective values in the condition that yielded the highest level of effort discounting (100 words typed backwards immediately) and observed a nonsignificant correlation, *r*(70) = −.025, *p* = .838. Similarly, the correlation between subjective values in this condition and time to complete the backwards typing task was also nonsignificant, *r*(70) = −.155, *p* = .194. Thus, the finding that need for cognition predicted effort discounting appears not to have been driven by individual differences in task proficiency.

## Discussion

The current study brings new insight to previous findings that people progressively devalue monetary rewards as the effort required to obtain them increases (i.e., “effort discounting”; Westbrook et al., 2013), concretely operationalizing (in monetary terms) the degree to which effort discounting diminished when people imagined applying cognitive effort in the future rather than imminently. As the temporal distance to future effort increased, not only did the absolute magnitude of effort discounting decrease, but so did sensitivity to gradations in effort level. Furthermore, not only did need for cognition inversely correlate with effort discounting overall (consistent with Westbrook et al., 2013), but individual differences in need for cognition modulated the impact of temporal distance on effort discounting: individuals high in need for cognition engaged in relatively little effort discounting regardless of temporal distance, whereas those low in need for cognition discounted cognitive effort progressively less the further in the future the effort lay, with an imagined month’s delay leading them to more closely resemble those high in need for cognition.

The finding that people discount cognitive effort less when the effort lies in the future could account for commonly experienced self-regulatory failures (also see Augenblick et al., 2015). For example, procrastination has been largely understood within the framework of delay discounting, occurring because distant rewards are discounted until a deadline approaches and rewards become more imminent (Steel & König, 2006). However, the current findings suggest that procrastination might additionally be framed in terms of dynamic inconsistency of effort-related decisions. Discounting future effort less than immediate effort conceivably could drive tendencies to undertake easier tasks in the present and save harder tasks for later. Adding to this, the present study suggests systematic individual differences in dynamic inconsistency, with individuals low in need for cognition more sensitive to the temporal distance of effort than those high in need for cognition. These findings, when considered alongside previous research showing a negative correlation between need for cognition and procrastination, offer further support for the prospect that dynamic inconsistency in effort discounting is connected to procrastination (Sarmány Schuller, 1999).

Deeper understanding of effort discounting, the impact of temporal distance, and such individual differences could suggest paths towards mitigating self-destructive tendencies to avoid cognitive effort. For example, it may be effective for some individuals (e.g., those low in need for cognition) to precommit to cognitively effortful tasks well in advance. The current findings suggest that such strategies may ameliorate differences in goal-directed behaviour between those who are averse to effort and those who enjoy it.

Although suggestive, there are some factors that may limit the generalizability of the present findings. The age and sex of participants were not recorded, so the possibility remains that these variables could have operated as unrecognized moderators. The participants were also all university students, so future work must assess whether similar patterns emerge among people who have not self-selected for pursuing cognitive challenge as a core feature of day-to-day life. It is also noteworthy that effort discounting was assessed specifically in relation to *cognitive* challenge. It may be that results differ when participants anticipate *physical* challenge, attitudes towards which also have significant health implications (e.g., Warburton et al., 2006). It is also worth noting that as anticipated effort levels increased in the current task, so did anticipated time devoted to the task (as often occurs in situations where task difficulty increases). It may be useful for future research to assess whether the current patterns hold when effort level is increased while holding anticipated task duration constant (but see Westbrook et al., 2013, for evidence of monotonic increases in effort discounting even when time between effort levels was equated).

## Conclusion

People often find the prospect of cognitive effort so displeasing that some have been found to prefer the experience of physical pain instead (Vogel et al., 2020). This can result in procrastination or avoidance of tasks that are consequential for success in many domains. Yet not all prospects of cognitive effort are equivalent. The current findings quantifiably demonstrate that effort discounting (i.e., the devaluing of an outcome in the face of effort) decreases the further into the future the effort lies. We found that this effect emerged more strongly among people who reported taking relatively little joy in cognitive challenge, with the results suggesting that their effort-based decisions may come to resemble those by people who revel in cognitive challenge when planning far enough into the future. Such patterns may suggest strategies that can modulate effort discounting in service of more effective decision making that consequently facilitates goal achievement in the domains of mental, financial, and possibly physical health.

## Footnotes

1 Note that this correlation collapsing across temporal distance and effort levels risks confounding delay discounting with effort discounting. Thus, we also assessed correlations between need for cognition and subjective values in the zero-delay condition, separately for 25, 50, 75, and 100 words typed backwards. Each of these were robust, respectively yielding: *r*(70) = .33, *p* = .005; *r*(70) = .34, *p* = .003; *r*(70) = .29, *p* = .014; and *r*(70) = .31, *p* = .007.

2 Consistent with this, Pearson correlations incorporating all participants revealed that need for cognition correlated with subjective values in the 100-word condition when effort was anticipated immediately, *r*(70) = .31, *p* = .007, and in a day, *r*(70) = .31, *p* = .009, but not in a month, *r*(70) = .13, *p* = .281.
